# Improved non-invasive positron emission tomographic imaging of chemotherapy-induced tumor cell death using Zirconium-89-labeled APOMAB®

**DOI:** 10.1186/s41181-020-00109-6

**Published:** 2020-11-17

**Authors:** Vasilios Liapis, William Tieu, Stacey E. Rudd, Paul S. Donnelly, Nicole L. Wittwer, Michael P. Brown, Alexander H. Staudacher

**Affiliations:** 1 Translational Oncology Laboratory, Centre for Cancer Biology, SA Pathology and University of South Australia, Adelaide, SA 5000 Australia; 2 School of Medicine, University of Adelaide, Adelaide, SA 5000 Australia; 3 Molecular Imaging and Therapy Research Unit (MITRU), South Australian Health and Medical Research Institute (SAHMRI), Adelaide, Australia; 4 School of Chemistry and Bio21 Molecular Science and Biotechnology Institute, University of Melbourne, Melbourne, Victoria 3010 Australia; 5 Cancer Clinical Trials Unit, Royal Adelaide Hospital, Adelaide, SA 5000 Australia

**Keywords:** Zirconium-89, Iodine-124, chDAB4, Chemotherapy, PET imaging

## Abstract

**Purpose:**

The chimeric monoclonal antibody (mAb) chDAB4 (APOMAB®) targets the Lupus associated (La)/Sjögren Syndrome-B (SSB) antigen, which is over-expressed in tumors but only becomes available for antibody binding in dead tumor cells. Hence, chDAB4 may be used as a novel theranostic tool to distinguish between responders and nonresponders early after chemotherapy. Here, we aimed to ascertain which positron emitter, Zirconium-89 ([^89^Zr]Zr^IV^) or Iodine-124 ([^124^I]I), was best suited to label chDAB4 for post-chemotherapy PET imaging of tumor-bearing mice and to determine which of two different bifunctional chelators provided optimal tumor imaging by PET using [^89^Zr]Zr^IV^-labeled chDAB4.

**Methods:**

C57BL/6 J mice bearing subcutaneous syngeneic tumors of EL4 lymphoma were either untreated or given chemotherapy, then administered radiolabeled chDAB4 after 24 h with its biodistribution examined using PET and organ assay. We compared chDAB4 radiolabeled with [^89^Zr] Zr^IV^ or [^124^I] I, or [^89^Zr]Zr-chDAB4 using either DFO-NCS or DFOSq as a chelator.

**Results:**

After chemotherapy, [^89^Zr]Zr-chDAB4 showed higher and prolonged mean (± SD) tumor uptake of 29.5 ± 5.9 compared to 7.8 ± 1.2 for [^124^I] I -chDAB4. In contrast, antibody uptake in healthy tissues was not affected. Compared to DFO-NCS, DFOSq did not result in significant differences in tumor uptake of [^89^Zr]Zr-chDAB4 but did alter the tumor:liver ratio in treated mice 3 days after injection in favour of DFOSq (8.0 ± 1.1) compared to DFO-NCS (4.2 ± 0.7).

**Conclusion:**

ImmunoPET using chDAB4 radiolabeled with residualizing [^89^Zr] Zr^IV^ rather than [^124^I] I optimized post-chemotherapy tumor uptake. Further, PET imaging characteristics were improved by DFOSq rather than DFO-NCS. Therefore, the radionuclide/chelator combination of [^89^Zr] Zr^IV^ and DFOSq is preferred for the imminent clinical evaluation of chDAB4 as a selective tumor cell death radioligand.

**Supplementary Information:**

**Supplementary information** accompanies this paper at 10.1186/s41181-020-00109-6.

## Introduction

Immuno-positron emission tomography/computed tomography (immunoPET/CT) is attractive clinically for the theranostic purpose of monitoring tumor responses to anticancer therapies because it enables the biodistribution and tumor targeting of mAbs to be quantified efficiently and with high spatial resolution. To allow time for specific tumor targeting and blood clearance, which maximizes tumor contrast against non-tumor background, a positron-emitting isotope with a half-life of 2–4 days is better suited for labeling mAbs because its physical half-life more closely matches the mAb’s biologic half-life, which may be days or weeks (van Dongen et al. 2007; Ovacik and Lin 2018). Consequently, the residualizing radiometal [^89^Zr] Zr^IV^ and the non-residualizing halogen [^124^I] I with physical half-lives of 3.27 and 4.18 days, respectively, are extensively used positron-emitters for clinical immunoPET studies (Jauw et al. 2016; Knowles and Wu 2012; Wright and Lapi 2013) and represent useful scouting matches for the long-lived therapeutic radionuclides (Verel et al. 2003).

Conventionally, therapy response monitoring relies on categorizing measurements of predefined changes in either tumor size (Eisenhauer et al. 2009) or tumor FDG uptake by PET (Wahl et al. 2009; Peacock et al. 2019). However, given that tumor cell death is a desired endpoint of anticancer therapies, specific and robust in vivo measures are being intensively investigated preclinically and clinically (Smith and Smith 2012; Zhang et al. 2019; Zhang et al. 2020). Among the cited in vivo tumor cell death detection technologies is the mAb DAB4.

The murine DAB4 mAb targets the abundant and ubiquitous nuclear RNA-binding protein, Lupus associated (La)/Sjögren Syndrome-B (SSB) antigen, which is overexpressed in a variety of cancers (Trotta et al. 2003; Sommer et al. 2011; Al-Ejeh et al. 2007a). However, La/SSB becomes available for specific antibody binding in cancer cells in vitro only after loss of plasma membrane integrity (i.e. in primary or secondary necrotic cancer cells) (Al-Ejeh et al. 2009a; Al-Ejeh et al. 2009b) and in vivo only after failed tumor clearance of post-apoptotic necrotic cells (Al-Ejeh et al. 2009a; Al-Ejeh et al. 2014), particularly after DNA-damaging anticancer treatment (Al-Ejeh et al. 2009a; Al-Ejeh et al. 2014). We have previously shown that following radiolabeling of DAB4 with ^111^In for tumor imaging (Al-Ejeh et al. 2007b), and ^90^Y (Al-Ejeh et al. 2009b), ^177^Lu (Staudacher et al. 2014a) and ^227^Th (Staudacher et al. 2014b) for tumor therapy, DAB4 binds with high specificity to dead tumor cells in vivo*.* Although these characteristics collectively make DAB4 a dead tumor cell radioligand, the technology of immunoPET facilitates longitudinal imaging and non-invasive biodistribution studies of DAB4 in tumor-bearing mice after chemotherapy, hence simulating a clinical scenario.

In this study, rather than murine DAB4, we have used the chimeric version of DAB4, chDAB4 (APOMAB®) (Staudacher et al. 2018; Staudacher  et al. 2020). In this format, the variable region sequences of murine DAB4 were genetically fused to the constant region sequences of human IgG1. Also, this human IgG1 Fc domain contains the K322A mutation, which is known to abrogate C1q binding and therefore complement-dependent cytotoxicity (Hezareh et al. 2001) (Liapis et al. submitted).

Given that a previous study had directly compared [^124^I] I or [^89^Zr] Zr^IV^ labeled versions of a mAb specific for an internalizing antigen (Cheal et al. 2014) and our own data had indicated that macrophage-mediated internalization of chDAB4 bound to dead tumor cells explained the anti-tumor activity of antibody drug conjugates of chDAB4 (Staudacher et al. 2018), we wished to compare the biodistribution and tumor uptake of chDAB4 mAb labeled with these two positron emitters.

The biodistribution of the radiolabeled chDAB4 was investigated using mice bearing syngeneic subcutaneous implants of the EL4 thymic lymphoblastic lymphoma. The mice were untreated or treated with DNA-damaging chemotherapy to induce tumor cell death including post-apoptotic necrosis (Al-Ejeh et al. 2009a; Al-Ejeh et al. 2014). The EL4 model was chosen because it has been well characterized by us (Al-Ejeh et al. 2009a) and others (Zhao et al. 2001) as a robust model of chemotherapy-induced apoptosis. Earlier, we had shown that the extent of post-chemotherapy tumor uptake of DAB4 predicted longer survival times of tumor-bearing mice, but this demonstration relied on invasive methods to determine the extent of tumor cell death (Al-Ejeh et al. 2009a). Here, we aim to validate these earlier findings by using chDAB4-immunoPET as a non-invasive method to show that post-chemotherapy tumor uptake of chDAB4 precedes tumor growth delay, which simulates the clinical scenario.

We also compared the commercially available bifunctional chelator H_3_DFO-*p*-phenyl-isothiocyanate (H_3_DFO-*p*-PhNCS or DFO-NCS), which has been used extensively in vivo both in non-clinical and clinical applications to conjugate mAbs for their subsequent radiolabeling with [^89^Zr] Zr^IV^ (Dmochowska et al. 2018; Hagens et al. 2018; Vosjan et al. 2010; Vugts et al. 2017; Dilworth and Pascu 2018), with our newly developed novel chelator H_3_DFOSqOEt (DFOSq), which aims to achieve more stable octadentate binding of [^89^Zr] Zr^IV^ by contributing oxygen molecules to the DFO chelator. DFOSq is a squaramide ester derivative of H_3_DFO-*p*-PhNCS and has previously been used to radiolabel the anti-HER2 antibody, trastuzumab, with [^89^Zr] Zr^IV^. The results of this study indicated that DFOSq had better stability in vivo and an improved tumor to background imaging quality than DFO-NCS (Rudd et al. 2016). In addition, recent data indicate that DFOSq has an improved shelf life compared to DFO-NCS (Berg et al. 2020). Hence, since we intended to employ DFOSq as the bifunctional chelator for conjugating chDAB4 in a future clinical study, we wished to test its attributes for this task in comparison to the industry standard, DFO-NCS.

In the current preclinical immunoPET study of a ligand that preferentially binds dead tumor cells, we wished to select the optimal positron emitter for imaging tumor cell death in vivo and to select a bifunctional chelator for [^89^Zr] Zr^IV^ to take forward in clinical PET imaging studies. We provide real-time imaging data in favour of chDAB4’s utility as a selective marker of chemotherapy-induced tumor cell death. Given that malignant rather than normal healthy tissues were specifically targeted after chemotherapy, these results support the clinical development of radiolabeled chDAB4 as a theranostic imaging agent with the potential to deliver dosimetric data in a clinical setting.

## Material and methods

### Cell culture and antibodies

EL4 murine thymic lymphoma cells were obtained from American Type Cell Culture (ATCC) and cultured in RPMI1640 containing 10% fetal calf serum (FCS) (Bovogen Biologicals, Victoria, Australia) with streptomycin and penicillin (Sigma-Aldrich). Cells were negative for mycoplasma contamination using MycoAlert® Mycoplasma Detection Kit (Lonza, Basel, Switzerland). The chimeric version of DAB4 (chDAB4) was created at CSIRO Molecular and Health Technologies (Parkville, VIC, Australia) by genetically fusing the variable region sequences of murine DAB4 to the constant region sequences of human IgG1. Using the CHO-XL99 expression system, a stable producer cell line of chDAB4 was manufactured at the National Biologics Facility, Australian Institute for Bioengineering and Nanotechnology, University of Queensland (Brisbane, QLD, Australia) (Staudacher et al. 2018; Staudacher et al. 2020).

### La ELISA and Lindmo binding assay

The binding of chDAB4 to the previously published La/SSB epitope (Tran et al. 2002) was performed as described (Staudacher et al. 2018). The Lindmo binding assay (Lindmo et al. 1984) was used to determine the immunoreactive fraction (IRF) of radiolabeled preparations of chDAB4. EL4 lymphoma cells were fixed and permeabilized using 10% neutral buffered formalin and methanol as previously described (Al-Ejeh et al. 2009a; Al-Ejeh et al. 2009b), resuspended in PBS with 1% FCS and serially diluted in duplicate 0.5 mL aliquots at cell numbers ranging from 5.5 × 10^7^ to 3.4 × 10^6^. Then 0.5 mL of 50 ng/mL radiolabeled antibody in 1% FCS was added to the cell suspensions. After incubation at 4 °C overnight, cells were centrifuged at 300 *g,* and 0.5 mL of the supernatant from each sample was placed in a separate tube and the radioactivity in the cell pellets and the supernatant was measured using the Hidex Automatic Gamma Counter.

### Conjugation and radiolabeling of chDAB4 with [^89^Zr] Zr^IV^ and [^124^I]I

chDAB4 was conjugated to the bifunctional chelator H_3_DFO-*p*-PhNCS (DFO-NCS) (Macrocyclics, Texas, USA) as previously described (Vosjan et al. 2010; Holland et al. 2010). The bifunctional chelator H_3_DFOSqOEt (DFOSq), which is a squaramide ester derivative of DFO, was synthesized and conjugated to the antibody as described (Rudd et al. 2016). The chelator to antibody ratio was determined by Electro Spray Ionisation Mass Spectrometry (ESI-MS) on an Agilent 6510 ESI-TOF LC/MS Mass Spectrometer (Agilent, California USA) and was 1.3:1 for DFO-NCS and 2.55:1 for DFOSq. All data were acquired and reference mass corrected via a dual-spray electrospray ionisation (ESI) source. [^89^Zr] Zr^IV^ oxalate was produced via proton irradiation of a ^89^Y target on a PETtrace 880 cyclotron (GE Healthcare) and purified on an Alceo purification system (Comecer, Italy) in the Molecular Imaging and Therapy Research Unit (MITRU) of the South Australian Health and Medical Research Institute (SAHMRI) as described (Holland et al. 2009) and the immunoconjugates were radiolabeled as described previously (Vosjan et al. 2010). Briefly, while gently shaking at room temperature, 90 μL of 2 M Na_2_CO_3_ was added to 120 MBq of [^89^Zr] Zr^IV^ oxalate solution and incubated for 3 min at room temperature, 300 μL of 0.5 M HEPES buffer and 1 mg of chDAB4 conjugated to DFO-NCS in 0.25 M NaCO_3_ were then added, followed by an additional 700 μL of 0.5 M HEPES buffer (pH 7.1) and incubated for 1 h at room temperature.

Sodium Iodide [^124^I] I in 0.02 M NaOH solution was purchased from Austin Health (Heidelberg, Vic, Australia). Conjugation of [^124^I] I to chDAB4 was performed using Pierce® Iodination beads (Thermo Scientific, Massachusetts, USA). Briefly, iodination beads were washed in PBS and allowed to dry before being added to 100 μL PBS and 100 MBq of [^124^I] I and incubated for 5 min at room temperature. One milligram of chDAB4 was added and incubated for 10 min at room temperature before the solution was removed from the beads.

Both [^89^Zr] Zr^IV^- and [^124^I] I-labeled antibodies were buffer-transferred and concentrated using Amicon® 50 kDa cut-off centrifugal filter devices (Millipore Massachusetts, USA) and resuspended in PBS for injection. Instant thin layer chromatography (ITLC) confirmed that < 1% free radionuclide was present in the preparations.

### Animal tumor inoculations and treatments

All animal experiments were approved by the SAHMRI Animal Ethics Committee (Adelaide, Australia), and were conducted following institutional ethical guidelines. Female C57BL/6 J mice 6–10 weeks old were inoculated subcutaneously in the right flank with 10^6^ syngeneic EL4 cells in PBS. Mice in the untreated group were injected with tumor cells 3 days later. This method of staggering tumor growth allows size-matched tumors to be compared at the time of imaging of the animals, thus mimicking clinical practice (Al-Ejeh et al. 2009a; Al-Ejeh et al. 2009b). Electronic calipers were used to measure the dimensions of the tumor, the volume of which was calculated as (a^2^ × b)/2, where a is the shortest diameter and b is the longest diameter of the tumor. Mice with 7-day old tumors were given intraperitoneal injections of 25 mg/kg cyclophosphamide and 19 mg/kg etoposide (Day 0). The following day mice were given intravenous injections with 5 MBq/50 μg of [^124^I] I- or [^89^Zr] Zr^IV^-labeled chDAB4 using the commercially available chelator DFO-NCS. To compare the different bifunctional chelators, 6 MBq/18 μg of [^89^Zr] Zr^IV^-labeled chDAB4, which had been conjugated either with DFO-NCS or DFOSq, was given intravenously.

### Stability of radiolabeled antibodies

To test in vitro stability of the radiolabeled antibodies, 500 kBq of the radiolabeled antibody was incubated in FCS at 37 °C for 6 days following radiolabeling. Samples were analyzed by ITLC using 20 mM citric acid (pH 5) as the solvent.. ITLC strips were cut in half and analyzed using the Hidex gamma-counter to measure the amount of free [^89^Zr] Zr^IV^ or [^124^I] I present.

### Animal imaging

For PET imaging, mice were gas-anesthetized with isoflurane (Veterinary Companies of Australia, NSW, Australia) and scanned for 10 min using the Albira Si PET-SPECT small animal scanner (Bruker Biospin GmbH, Valencia, Spain). Regions of interest were manually drawn around the tumor using PMOD® software (PMOD Technologies, Switzerland) and the tumor uptake determined as the injected dose per gram (%ID/g) following decay correction as calculated by the PMOD software. Area under-curve (AUC) values were calculated from activity curves, which were generated over 8 days.

At the end of the experiment, organs were removed, weighed and the accumulation of [^89^Zr] Zr^IV^ and [^124^I] I was measured using the Hidex gamma-counter. Radioactivity in the organs was normalized to organ weight and calculated as the percentage of radioactivity per gram over the radioactivity of the injected dose of the radiolabeled chDAB4 (%ID/g). Tumor to organ ratios were calculated by dividing the %ID/g values for tumor by those for blood, bone or liver.

### Statistical analysis

Statistical analyses were performed using GraphPad Prism (v8.0) software. Comparison of two groups was performed by two-tailed *t*-test or intergroup comparisons made by two-way analysis of variance (ANOVA). Data are shown as the mean ± standard deviation (SD). Statistical significance was reached when *p* < 0.05, with * representing p < 0.05, ** *p* < 0.01, *** *p* < 0.001 and **** *p* < 0.0001.

## Results

### Stability and antigen binding of radiolabeled chDAB4 in vitro

**Fig. 1:**
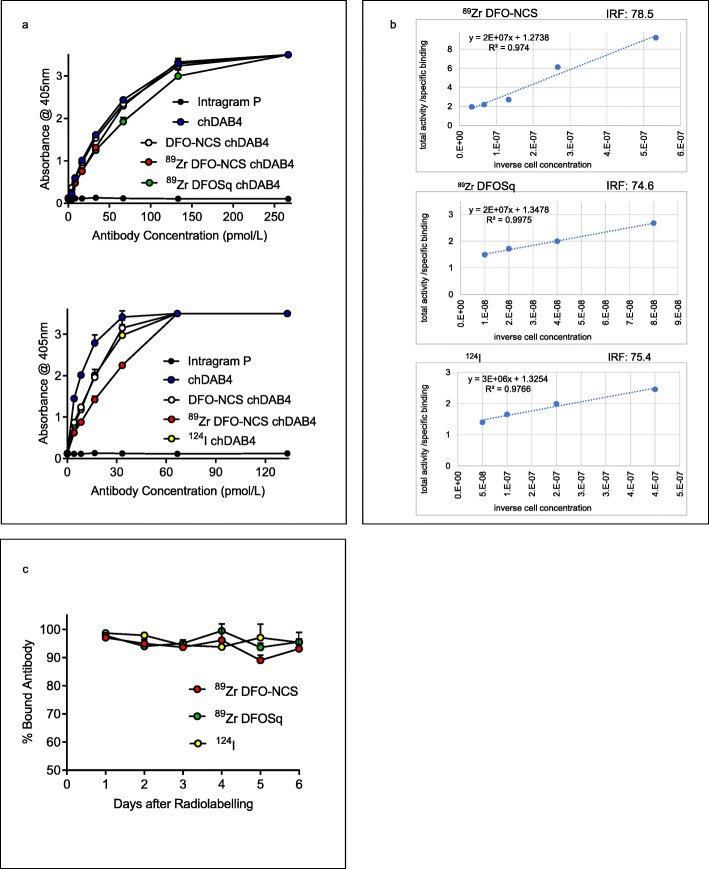
Immunoreactivity and stability of conjugates and radioconjugates of chDAB4. Binding of chDAB4 to the La peptide was measured by ELISA and is equivalent for DFO-NCS-chDAB4, [^89^Zr]Zr-DFO-NCS-chDAB4, [^89^Zr]Zr-DFOSq-chDAB4, and [^124^I]I-labeled chDAB4 (*n* = 3) (**a**). Lineweaver-Burk plots show values for the immunoreactive fraction (IRF) determined by the Lindmo assay. The IRF values for the [^89^Zr]Zr-labeled DFO-conjugates of chDAB4, [^89^Zr]Zr-DFO-NCS and ^89^Zr-DFOSq, and for [^124^I]I-labeled APOMAB (^124^I), were 78.5%, 74.6% and 75.4%, respectively (**b**). The chDAB4 mAb radiolabeled with [^89^Zr] Zr^IV^ or [^124^I] I is stable over 6 days in FCS at 37 °C as determined by ITLC (**c**). All data points are means ± SD

Radiolabeling of DFO-NCS-conjugated chDAB4 with [^89^Zr] Zr^IV^ or radiolabeling of intact chDAB4 with [^124^I] I resulted in specific activities of ≥100 MBq/mg with < 1% free radionuclide in each preparation. ELISA was used to compare binding to the La-derived peptide epitope of unconjugated chDAB4, DFO-conjugates of chDAB4, and radiolabeled versions of the chDAB4 conjugates. All antibodies bound with high avidity to the La peptide with dissociation constants (Kd ± SEM) of 32.1 pmol/L ± 4.8 for chDAB4, 33.8 pmol/L ± 4.9 for DFO-NCS-chDAB4, 38.2 pmol/L ± 7.3 for [^89^Zr]Zr-DFO-NCS-chDAB4 and 46.2 ± 7.7 for [^89^Zr]Zr-DFOSq-ch-DAB4 (Fig. 1a. top panel). In a separate ELISA experiment, we compared the Kd values for [^124^I]I-and [^89^Zr]Zr^IV^-radiolabeled chDAB4 mAbs. The Kd (± SEM) values were 4.9 ± 1.0, 10.2 ± 2.9, 10.8 ± 3.1 and 16.9 ± 5.5 pmol/L for chDAB4, DFO-NCS-chDAB4, and chDAB4 radiolabeled with [^124^I] I and [^89^Zr] Zr^IV^, respectively (Fig. 1a. bottom panel). The immunoreactive fraction (IRF) was also determined for each form of chDAB4 by the Lindmo assay, with the IRF (± SD) for [^89^Zr]Zr-DFO-NCS being 78.5 ± 9.1%, 74.6 ± 0.8% for [^89^Zr]Zr-DFOSq-ch-DAB4 and 75.4 ± 10.1% for [^124^I]I-chDAB4, demonstrating that the conjugation and radiolabeling procedures had little effect on the immunoreactivity of the chDAB4 radioconjugates (Fig. 1b).

Radiolabeled antibodies were stable in FCS at 37 °C during a 6-day period in vitro: free [^89^Zr] Zr^IV^ was detected in the range of 0.5–10.9% and free [^124^I] I was detected in the range of 1.4–6.3% (Fig. 1c).

### The chDAB4 mAb radiolabeled with [^89^Zr] Zr^IV^ binds to dead tumor cells after chemotherapy in vivo

**Fig. 2:**
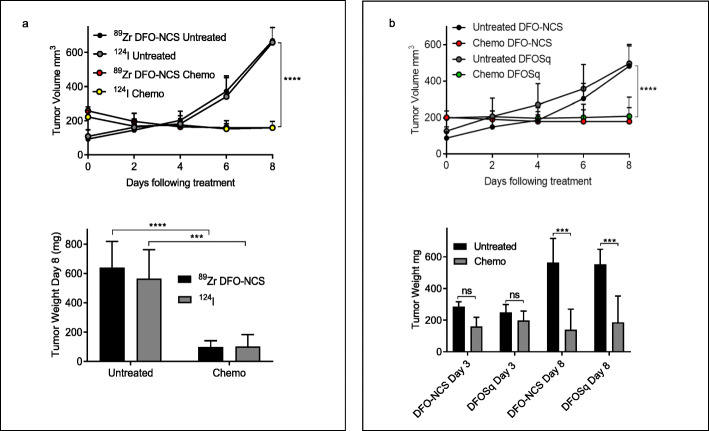
Effects of chemotherapy on tumor size in mice administered different radioconjugates of chDAB4. Tumor growth curves and tumor weights (*n* = 5) testing different radiolabels (**a**) or different bifunctional chelators (**b**). All data points are means ± SD

As seen in previous studies (Al-Ejeh et al. 2009a; Al-Ejeh et al. 2014; Staudacher et al. 2014a; Staudacher et al. 2014b; Staudacher et al. 2018), EL4 tumors hosted by syngeneic mice were chemo-responsive resulting in significant reductions in tumor size determined by caliper measurements and ex vivo tumor weights (Fig. 2a and b). As expected, no significant differences were observed in tumor growth rates between mice receiving [^89^Zr]Zr^IV^- or [^124^I]I-labeled antibodies alone or after chemotherapy, indicating that the radionuclides per se had no appreciable therapeutic effect (Fig. 2).

**Fig. 3:**
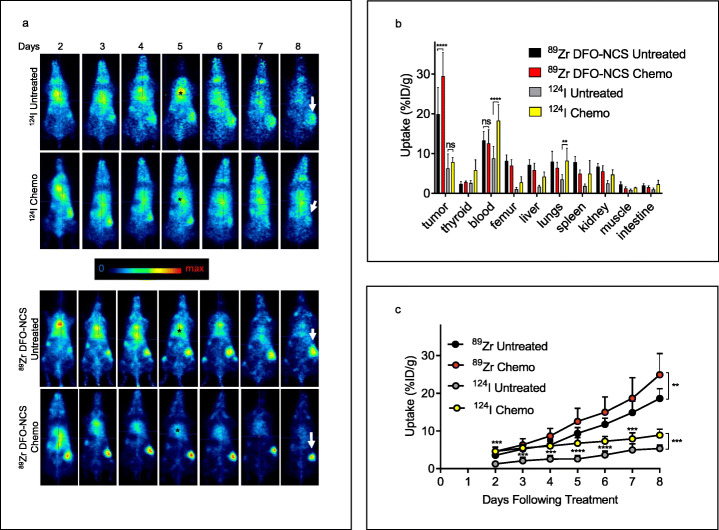
Effect of chemotherapy on tumor and normal tissue uptake of different radioconjugates of chDAB4. Representative whole-body PET images of a single mouse from each group during the course of the experiment are shown. Color scale indicates relative extent of tissue uptake of radioactivity. Asterisks in black at Day 5 time point indicate blood pool. Arrows in white at Day 8 time point indicate tumors in right flank of each mouse (n = 5) (**a**). Ex vivo biodistribution of the [^89^Zr]Zr^IV^- and [^124^I]I-labeled chDAB4 on Day 8 was measured using the HIDEX counter (**b**). Tumor uptake of [^124^I]I- and [^89^Zr]Zr^IV^-labeled chDAB4 during the course of the experiment was quantified and expressed as Percent Injected Dose per gram (%ID/g) using PMOD® software (**c**). All data points are means ± SD

Next, we examined the biodistribution of [^89^Zr]Zr-chDAB4 and [^124^I]I-chDAB4 in tumor bearing mice (Fig. 3). Tissue biodistribution showed significant blood and lung accumulation of [^124^I] I activity in treated mice (mean %ID/g ± SD; 18.3 ± 4.0 and 8.2 ± 3.1) compared to untreated mice (8.8 ± 2.9 and 3.5 ± 1.2), respectively. Notwithstanding the availability of [^124^I]I-chDAB4 in serum, relatively little [^124^I]I-chDAB4 had accumulated in tumors at the end of the experiment (Fig. 3b), although PMOD analysis shows significantly more tumor accumulation of [^124^I]I-chDAB4 in treated mice over the course of the experiment (Fig. 3c). In contrast, in mice administered [^89^Zr]Zr-chDAB4, significant tumor accumulation of tracer after chemotherapy was evident (± SD): 19.9 ± 6.7 vs 29.4 ± 5.8%ID/g for untreated vs treated mice (Fig. 3b), with no significant differences in normal tissues. Furthermore, PMOD analysis demonstrated that chemotherapy resulted in incremental tumor accumulation of [^89^Zr]Zr-chDAB4 (Fig. 3c).

Using decay-corrected PMOD data over the course of the experiments, we estimated that cumulative exposure of post-chemotherapy tumors to the activity (%ID/g ± SD) of [^89^Zr]Zr-chDAB4 compared to that of [^124^I]I-chDAB4 was 75.9 ± 14.3%ID/g per hour vs 40.1 ± 5.2%ID/g per hour, respectively.

In summary, these data indicate that the [^89^Zr] Zr^IV^ label residualizes in tumors in contradistinction to the [^124^I] I label.

### Comparison of biodistribution of chDAB4 conjugated with DFO-NCS or DFOSq

**Fig. 4:**
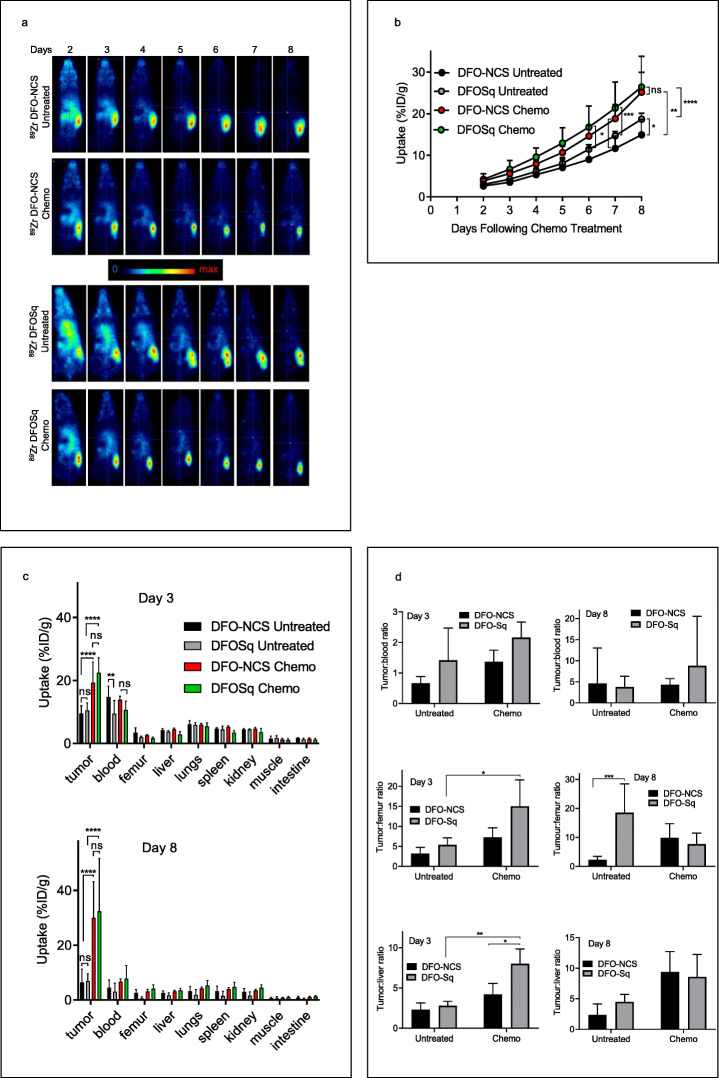
Effects of chemotherapy on tumor and normal tissue uptake of [^89^Zr]Zr^IV^-labeled chDAB4 using different bifunctional chelators. Representative whole-body PET images of a single mouse from each group (n = 5) during the course of the experiment are shown. Color scale indicates relative extent of tissue uptake of radioactivity. Arrows in white at Day 8 time point indicate tumors in right flank of each mouse (**a**). Tumor uptake of the [^89^Zr]Zr^IV^-labeled antibodies during the course of the experiment was quantified and expressed as Percent Injected Dose per gram (%ID/g) using PMOD® software (**b**). The biodistribution of [^89^Zr]Zr^IV^-labeled DFO-NCS (DFO) and DFOSq conjugates of chDAB4 was measured using the HIDEX counter in tumor and organs taken from mice on Days 3 and 8 **(c)**. On Day 3, %ID/g derived from physical γ-counts measured in different organs were expressed as a ratio for tumor to blood, tumor to bone, or tumor to liver (**d**). All data points are means ± SD

Next, we compared the two different types of bifunctional chelator, DFO-NCS and DFOSq, for the labeling of chDAB4 with [^89^Zr]Zr^IV^. Representative PET images are shown in Fig. 4a. Using organ assays to measure physical γ-counts, tumor accumulation of [^89^Zr]Zr-chDAB4 was significantly greater in treated mice compared to untreated mice regardless of whether chDAB4 was conjugated with DFO-NCS or DFOSq (Fig. 4a and b). On Day 3, mean tumor uptake (%ID/g ± SD) of [^89^Zr]Zr^IV^-labeled DFO-NCS- and DFOSq-conjugates of chDAB4 was 19.3 ± 6.6 and 22.5 ± 4.8 in treated mice vs 9.5 ± 2.5 and 10.5 ± 2.4 in untreated mice, respectively. On Day 8, mean tumor uptake (%ID/g ± SD) was 30.0 ± 13.2 and 32.4 ± 19.3 in treated mice vs 6.5 ± 4.9 and 6.9 ± 2.7 in untreated mice, respectively. Although we did not observe any significant differences in tumor uptake of [^89^Zr]Zr-chDAB4 using either chelator in separate studies on Days 3 or 8 we did observe significantly accelerated blood clearance of the [^89^Zr]Zr-labeled DFOSq-conjugate of chDAB4 compared to the DFO-NCS-conjugate on Day 3 in untreated mice (14.8 ± 3.4 vs 9.5 ± 4.1%ID/g ± SD) (Fig. 4c). On the other hand, image processing and volume of interest (VOI) analysis using PMOD software over the 8-day scanning period showed significantly greater tumor uptake of [^89^Zr]Zr-chDAB4 in untreated mice using the DFOSq-conjugate compared to the DFO-NCS-conjugate (Fig. 4b).

It is important to note that organ assays did not demonstrate any significant differences in normal tissue uptake including bone of [^89^Zr]Zr-chDAB4 irrespective of chemotherapy use or the type of bifunctional chelator (Fig. 4c). However, by comparing the physical γ-counts in the organ assays done on Day 3, we calculated the ratio of %ID/g in tumor to that in blood, bone (femur) or liver for each type of chelator. Compared to the standard DFO-NCS-conjugate, there was a significantly higher tumor:tissue ratio for bone and liver for the DFOSq-conjugate (Fig. 4d). The average tumor to bone ratio for the DFOSq-conjugate was 15.0 vs 5.3 in treated vs untreated mice, which suggests that the DFOSq-conjugate may be more stable and thus less likely to release [^89^Zr] Zr^IV^, which targets bone (Fischer et al. 2013). The average tumor to liver ratios for the DFOSq-conjugate were 8.0 vs 2.8 in treated vs untreated mice and 8.0 vs 4.2 in treated mice that received the DFOSq-conjugate vs the DFO-NCS-conjugate. These results suggest that the DFOSq-conjugate of [^89^Zr]Zr-chDAB4 may yield better imaging quality than the DFO-NCS-conjugate. By Day 8, no significant differences were observed in the tumor: normal tissue ratios irrespective of the type of chelator with average tumor to liver ratios for the DFOSq-conjugate of 4.5 vs 8.6 in treated vs untreated mice and 9.3 vs 8.6 in treated mice that received the DFOSq-conjugate vs the DFO-NCS-conjugate.

## Discussion

To determine the preferred positron emitter for the planned clinical PET detection of chemotherapy-induced tumor cell death and to gain a better understanding of the processing of radiolabeled chDAB4 within tumors, we labeled chDAB4 with residualizing [^89^Zr] Zr^IV^ or non-residualizing [^124^I] I. We observed pronounced tumor uptake and retention of [^89^Zr]Zr^IV^-labelled chDAB4 compared to [^124^I]I-labelled chDAB4, particularly when given after chemotherapy. Interestingly, these results match those seen by others who used internalizing antibodies labeled with [^89^Zr] Zr^IV^ and [^124^I] I, and showed that [^89^Zr] Zr^IV^ is trapped within the cells whereas [^124^I] I is released from cells (Wu 2014; Mendler et al. 2015; Fung et al. 2016; Deri et al. 2013). For example, in a pairwise comparison of [^124^I]I- and [^89^Zr] Zr^IV^ labeled versions of girentuximab, which is an internalizing mAb specific for carbonic anhydrase 9, both antibody preparations had equivalent in vivo tumor uptake kinetics in a xenograft model of human clear cell renal cell carcinoma but only [^89^Zr]Zr^IV^-girentuximab resulted in prolonged tumor retention (Cheal et al. 2014).

However, given that the La/SSB target antigen of the radiolabeled chDAB4 mAb is revealed preferentially in dead tumor cells in vivo *(*Al-Ejeh et al. 2009a*;* Al-Ejeh et al. 2014*;* Staudacher et al. 2014a*;* Staudacher et al. 2014b*;* Staudacher et al. 2018*)*, the antibody radioconjugate will not be further processed unless it is by a non-targeted viable cell type. We hypothesize that tumor-associated macrophages (TAMs), which are highly abundant within tumor necrotic regions (Murdoch et al. 2004) and further enriched after chemotherapy (Staudacher et al. 2018), engulf and process [^89^Zr]Zr-chDAB4 bound dead tumor cells.

This TAM-mediated internalization of chDAB4-bound tumor cells, particularly after chemotherapy, may thus enable [^89^Zr] Zr^IV^ to be residualized and would explain why significantly more [^89^Zr]Zr-chDAB4 was detected in the tumor compared to [^124^I]I-chDAB4. On the other hand, as a non-residualizing radionuclide, ^124^I would be released extracellularly and may thus account for the higher ^124^I activity found in blood and lung (as blood pool) after chemotherapy. As we described for macrophage-mediated processing of DAB4 antibody drug conjugates in the syngeneic Lewis lung carcinoma model in which C57BL/6 J mice bear subcutaneous tumors of LL2 cells (Staudacher et al. 2018), TAM-mediated phagocytosis of [^89^Zr]Zr-chDAB4-bound dead tumor cells may account for the post-chemotherapy finding of augmented tumor uptake of [^89^Zr]Zr-chDAB4 in the EL4 lymphoma model. This EL4 model is well-characterized and the La/SSB-specific mAb binds specifically, rapidly, avidly, and irreversibly to the La/SSB target antigen in post-apoptotic necrotic EL4 tumor cells (Al-Ejeh et al. 2009a; Al-Ejeh et al. 2009b).

Work published by Donnelly et al. compared their novel chelator DFOSq to DFO-NCS using the HER2-targeting antibody, trastuzumab (Rudd et al. 2016). In mice bearing HER2-positive human ovarian cancer and breast cancer xenografts, as measured by ratios of tumor to background, bone and liver and by tumor SUV_max_ measurements according to type of chelator, [^89^Zr]Zr-DFOSq–trastuzumab showed improved PET imaging and radiolabeling efficiency over DFO-NCS, thus warranting further investigation. Although we consistently observed numerically higher %ID/g values over the course of the experiment for tumor uptake of [^89^Zr]Zr-DFOSq-chDAB4 compared to [^89^Zr]Zr-DFO-NCS-chDAB4, either by organ assay or PMOD analysis, statistical significance was not reached. However, 3 days after chemotherapy and before tumor growth delay is evident (Fig. 3) we did identify improved tumor to normal tissue ratios for [^89^Zr]Zr-DFOSq-chDAB4 compared to [^89^Zr]Zr-DFO-NCS-chDAB4. These data indicate that the post-chemotherapy tumor uptake of [^89^Zr]Zr-DFOSq-chDAB4 significantly exceeded its uptake in both bone and liver, unlike [^89^Zr]Zr-DFO-NCS-chDAB4. As an example of determining optimal tumor to nontumor ratios, the liver has been the most studied and extensively used reference region for clinical whole-body FDG-PET (Hofheinz et al. 2016).

Contrary to other [^89^Zr] Zr^IV^ antibody radiolabeling studies [29, 30] [^89^Zr]Zr-chDAB4 showed high stability in vitro and the relatively low bone uptake compared to tumor uptake also indicated in vivo stability over 7 days. PET quantification imaging and biodistribution data indicate free [^89^Zr] Zr^IV^ accumulation in the bone is minimal when compared to radiolabeled antibody uptake in the tumor. Antibody accumulation in healthy tissues was further reduced when injected amounts of radiolabeled chDAB4 were reduced from 50 μg (Fig. 3) to 18 μg (Fig. 4), leading to higher tumor to background contrast, in agreement with specific and saturable in vivo tumor binding of the antibody (Al-Ejeh et al. 2007b).

## Conclusion

Here, we show that chDAB4 labeled with a positron-emitting radionuclide is stable both in vitro and in vivo and enables whole-body live animal PET imaging of chemotherapy-induced tumor cell death, which subsequently manifests as tumor growth delay. Consequently, immunoPET with chDAB4 might be employed clinically to discern in whom the cancer will shrink or stabilize soon after a first course of DNA-damaging chemotherapy. However, the ability of chDAB4 to serve this purpose effectively depends on the specific positron-emitter used. ImmunoPET with [^124^I]I-chDAB4 displays low tumor uptake and could not adequately discriminate the effect of chemotherapy on tumor cell death. In contrast, immunoPET with [^89^Zr]Zr-chDAB4 shows high and persistent tumor accumulation of the radiolabel after chemotherapy, presumably because of the residualizing properties of [^89^Zr] Zr with or without its chelator.

Moreover, we found that [^89^Zr]Zr-labeling of the chDAB4 immunoconjugate employing the squaramide ester form of DFO rather than commercially available DFO-NCS form resulted in superior imaging characteristics, which may be better suited to its application in clinical immunoPET. In this respect, we have commenced an IRB-approved phase 1 clinical theranostic study of [^89^Zr]Zr-DFOSq-chDAB4 in patients with advanced lung and ovarian cancer after they receive their first cycle of platinum-based chemotherapy (Australian and New Zealand Clinical Trials Registry No. ACTRN12620000622909). Using this radiotheranostic agent to visualize and quantify chemotherapy-induced tumor cell death may enable immunoPET to distinguish chemosensitive from chemoresistant types of cancer.

## Supplementary Information

**Additional file 1.**

## Data Availability

The datasets generated during and/or analysed during the current study are available from the corresponding author on reasonable request.

## Abbreviations

ANOVA: Analysis of variance
ATCC: American Type Cell Culture
chDAB4: Chimeric DAB4
DFO-NCS: H3DFO-*p*-phenyl-isothiocyanate
DFOSq: H3DFOSqOEt
ESI-MS: Electro spray ionisation mass spectrometry
FCS: Fetal calf serum
immunoPET/CT: Immuno-positron emission tomography/computed tomography
IRF: Immunoreactive fraction
^111^In: Indium-111
%ID/g: % injected dose per gram
ITLC: Instant thin layer chromatography
[^124^I]I: Iodine-124
La/SSB: Lupus-associated or Sjögren Syndrome-B antigen
^177^Lu: Lutetium-177
MITRU: Molecular Imaging and Therapy Research Unit
SAHMRI: South Australian Health and Medical Research Institute
SD: Standard deviation
SEM: Standard error of the mean
SUV: Standardized uptake value
^227^Th: Thorium-227
TAMs: Tumor-associated macrophages
VOI: Volume of Interest
^90^Y: Yttrium
[^89^Zr]Zr^IV^: Zirconium-89
