# Supplementary material for: Improved non-invasive positron emission tomographic imaging of chemotherapy-induced tumor cell death using Zirconium-89-labeled APOMAB®
Source: EJNMMI Radiopharm Chem. 2020 Nov 17;5:27. doi: 10.1186/s41181-020-00109-6 (PMC7672150; doi:10.1186/s41181-020-00109-6)
Supplement: Supplementary file 1 — Additional file 1. [file 41181_2020_109_MOESM1_ESM.pdf]

Supplementary Table.1

a

	^{89}Zr Untreated					^{89}Zr Chemo					^{124}I Untreated					^{124}I Chemo				
**Mouse**	**1**	**2**	**3**	**4**	**5**	**1**	**2**	**3**	**4**	**5**	**1**	**2**	**3**	**4**	**5**	**1**	**2**	**3**	**4**	**5**
Tumor	21.38	17.23	30.99	14.89	14.94	35.14	25.53	26.12	24.05	36.58	10.84	3.68	9.62	3.13	4.14	9.50	6.76	7.13	7.05	8.72
Thyroid	2.88	2.45	3.03	1.74	1.82	2.90	2.97	2.76	2.35	3.08	2.82	2.76	3.06	1.51	2.88	3.45	2.89	8.81	6.64	7.47
Blood	14.32	14.16	15.73	9.76	12.58	17.41	14.09	12.68	10.24	8.56	13.45	7.25	10.04	6.09	7.28	15.25	16.49	17.44	16.94	25.33
Femur	7.73	9.66	9.85	6.92	6.70	9.01	8.16	6.22	5.58	5.85	1.61	0.76	1.36	0.72	0.86	1.68	2.23	2.11	2.30	5.33
Liver	7.08	6.40	9.39	6.97	5.88	8.17	6.18	6.59	4.67	3.57	2.18	1.27	1.83	1.37	1.67	3.09	4.37	3.08	4.34	6.03
Lungs	7.37	7.28	12.41	5.64	7.25	7.71	7.67	6.83	5.12	4.65	5.09	2.60	4.40	2.50	3.05	6.24	6.91	7.92	6.41	13.73
Spleen	8.48	7.78	9.80	5.86	7.23	6.36	4.94	5.26	4.30	3.83	2.52	1.27	2.18	1.35	1.93	2.94	3.73	3.35	3.92	10.86
Kidney	7.10	6.92	7.71	5.60	6.22	6.92	6.29	6.42	4.15	3.77	3.75	2.07	2.66	1.82	2.14	3.85	4.17	4.47	4.38	6.82
Muscle	1.43	3.17	2.67	1.96	1.88	1.86	1.36	1.12	0.84	1.03	1.13	0.96	0.96	0.84	0.57	1.39	1.53	1.15	1.39	1.53
Intestine	2.12	2.06	2.57	1.38	1.82	1.84	1.98	1.52	1.27	1.10	1.39	0.72	1.40	0.73	0.71	1.67	1.94	1.77	1.90	4.16

b

	^{89}Zr Untreated					^{89}Zr Chemo					^{124}I Untreated					^{124}I Chemo				
**Days**	**mouse 1**	**mouse 2**	**mouse 3**	**mouse 4**	**mouse 5**	**mouse 1**	**mouse 2**	**mouse 3**	**mouse 4**	**mouse 5**	**mouse 1**	**mouse 2**	**mouse 3**	**mouse 4**	**mouse 5**	**mouse 1**	**mouse 2**	**mouse 3**	**mouse 4**	**mouse 5**
**2**	2.89	3.55	4.02	3.81	3.46	5.14	5.06	5.00	2.73	4.96	1.81	1.41	1.32	0.77	0.95	4.31	3.78	5.30	3.37	6.15
**3**	3.86	5.73	5.58	5.59	5.24	6.61	6.82	7.81	3.53	6.80	3.60	1.93	2.15	1.39	1.31	5.21	5.35	5.60	4.31	6.90
**4**	5.41	7.11	7.31	4.01	7.41	8.57	8.72	9.54	5.51	10.95	3.98	2.46	2.66	2.13	1.58	5.51	5.55	6.65	4.49	7.87
**5**	7.39	10.97	10.51	8.07	9.87	12.14	13.61	15.17	6.68	14.98	2.00	3.87	3.03	2.39	1.61	6.22	6.16	7.04	5.06	9.13
**6**	10.11	13.41	13.32	10.04	11.74	14.70	15.87	19.80	8.63	15.89	5.21	3.40	4.04	2.99	2.54	6.66	7.05	7.88	5.72	9.07
**7**	12.61	17.07	18.20	10.97	15.54	19.07	20.16	24.05	9.47	20.54	7.39	5.28	5.03	4.16	2.89	7.59	6.84	9.02	6.07	9.99
**8**	17.23	21.81	19.73	15.01	19.30	24.84	27.57	29.05	15.28	27.84	6.78	4.91	5.57	4.99	4.47	8.02	7.44	10.05	7.79	11.08

c

	DFO-NCS Untreated					DFOSq Untreated					DFO-NCS Chemo					DFOSq Chemo				
**Days**	**Mouse 1**	**Mouse 2**	**Mouse 3**	**Mouse 4**	**Mouse 5**	**Mouse 1**	**Mouse 2**	**Mouse 3**	**Mouse 4**	**Mouse 5**	**Mouse 1**	**Mouse 2**	**Mouse 3**	**Mouse 4**	**Mouse 5**	**Mouse 1**	**Mouse 2**	**Mouse 3**	**Mouse 4**	**Mouse 5**
**2**	2.91	3.12	1.43	2.87	2.74	2.81	3.75	2.59	2.63	3.13	4.86	3.46	4.27	3.25	3.34	6.44	2.73	3.74	3.94	4.20
**3**	3.64	4.22	2.14	4.10	3.46	3.75	4.91	4.58	3.98	4.01	6.53	5.11	6.11	4.49	5.88	10.39	5.68	5.23	5.89	5.94
**4**	6.01	6.77	2.92	6.37	4.57	5.71	7.13	6.22	5.62	5.87	8.66	7.69	8.73	6.45	7.89	13.18	7.54	8.35	9.23	9.63
**5**	7.79	9.24	3.21	8.87	6.06	7.76	9.69	7.85	6.96	7.86	11.46	10.69	12.28	8.22	10.63	19.27	10.09	10.90	12.16	12.32
**6**	9.75	11.84	4.96	10.71	7.74	10.19	13.19	11.46	11.00	11.42	14.58	14.87	18.21	10.88	14.50	25.58	12.99	13.59	16.39	15.19
**7**	12.45	15.09	7.65	13.33	9.78	13.67	16.50	14.57	14.42	14.20	19.90	19.13	22.98	13.59	18.37	32.14	16.92	17.06	21.04	19.46
**8**	16.07	18.83	9.74	17.37	12.58	17.05	20.96	18.62	18.22	18.59	25.80	26.88	28.78	16.76	27.51	39.13	21.53	21.11	25.69	24.62

d

**Day 3**	DFO-NCS Untreated			DFOSq Untreated			DFO-NCS Chemo			DFOSq Chemo		
**Mouse**	**1**	**2**	**3**	**1**	**2**	**3**	**1**	**2**	**3**	**1**	**2**	**3**
Tumor	12.20	7.32	9.06	11.52	7.79	12.28	23.83	11.77	22.28	21.45	27.66	18.25
Blood	13.38	12.38	18.73	11.69	12.13	4.69	14.91	12.63	14.11	13.46	10.71	7.93
Femur	2.85	5.18	2.33	2.45	1.93	1.67	2.88	2.65	2.50	2.14	1.23	1.46
Liver	3.80	4.74	4.28	3.51	3.55	4.23	4.43	4.35	4.95	3.66	3.05	2.01
Lungs	7.42	5.47	5.49	6.48	6.14	5.24	6.44	5.67	5.80	6.61	5.36	4.45
Spleen	4.29	4.72	5.04	3.81	3.80	5.73	5.51	5.43	4.92	3.98	3.68	2.69
Kidney	4.83	4.16	4.31	4.22	4.73	4.34	5.17	4.50	4.35	4.87	3.54	2.39
Muscle	2.46	1.01	1.14	1.73	2.44	0.88	1.56	0.99	1.49	1.27	1.56	0.71
Intestine	1.93	1.68	1.74	1.67	1.36	0.81	1.86	1.39	1.40	1.62	1.24	0.83

e

**Day 8**	DFO-NCS Untreated					DFOSq Untreated					DFO-NCS Chemo					DFOSq Chemo				
**Mouse**	**1**	**2**	**3**	**4**	**5**	**1**	**2**	**3**	**4**	**5**	**1**	**2**	**3**	**4**	**5**	**1**	**2**	**3**	**4**	**5**
Tumor	12.15	3.12	10.30	6.26	0.46	10.34	9.17	5.30	4.78	4.89	46.01	20.20	34.91	13.08	35.88	55.79	22.09	16.62	16.49	50.74
Blood	0.62	7.20	5.42	6.92	2.31	8.41	3.25	1.25	0.60	1.92	8.22	5.63	7.14	6.00	6.63	12.81	8.70	6.36	0.55	10.73
Femur	3.21	2.93	3.15	3.28	0.31	1.34	1.16	0.19	0.21	0.19	4.17	2.74	3.52	3.29	2.10	6.60	3.52	3.50	3.40	3.64
Liver	2.54	3.13	2.95	3.09	1.16	2.46	2.93	0.82	1.08	1.16	3.79	2.74	3.00	2.91	3.18	4.54	3.57	2.64	3.14	3.95
Lungs	3.77	3.98	3.43	4.74	0.45	4.25	3.63	0.42	0.51	0.65	5.12	3.78	4.30	4.04	3.91	6.79	4.71	3.72	3.94	7.64
Spleen	2.63	4.96	3.59	4.52	0.26	2.64	3.97	0.22	0.23	0.24	4.63	3.32	3.75	4.50	3.66	7.39	4.18	3.08	3.83	5.55
Kidney	3.62	3.19	3.59	3.56	0.68	3.28	3.01	0.57	0.53	0.73	3.97	3.31	3.92	3.42	2.85	6.25	4.33	3.32	4.02	4.63
Muscle	1.02	0.41	0.68	0.89	0.25	1.69	0.61	0.17	0.21	0.19	1.09	1.06	0.66	0.64	0.50	1.52	0.65	0.56	1.10	1.10
Intestine	0.64	1.42	1.04	1.06	0.19	0.95	0.56	0.16	0.12	0.19	0.89	1.45	1.09	1.20	0.83	1.79	1.34	1.07	0.73	1.37

Supplementary table 1:. Quantitative values are shown for in figures 3b **(a)**, 3c **(b)**, 4b **(c)** and 4c **(d)**
